# 

**Published:** 1881-01

**Authors:** 


A MONTHLY JOURNAL,

DEVOTED TO

### *MEDICAL, SURGICAL, OBSTETRICAL, DENTAL and HYGIENICAL SCIENCE.*

EDITED BY
HARVEY L. BYRD, A. M., M. D.,
AND
BASIL M. WILKERSON, D. D. S., M. D.

**"Altera poscit opem res, et conjurat amice."**

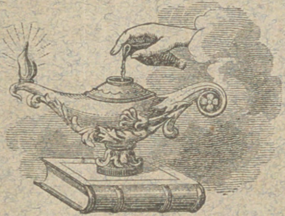

BALTIMORE:
*Published by B. M. WILKERSON, Proprietor,*
Office, 68 N. Charles Street.

$2.00 PER ANNUM IN ADVANCE. - - - - SINGLE COPIES 25 CENTS.

PRINTED BY THOMAS & EVANS, 9, 11, & 13 S. HOLLIDAY ST., BALTO.

